# Typhoid and Paratyphoid Cost of Illness in Bangladesh: Patient and Health Facility Costs From the Surveillance for Enteric Fever in Asia Project II

**DOI:** 10.1093/cid/ciaa1334

**Published:** 2020-12-01

**Authors:** Nelly Mejia, Sarah W Pallas, Samir Saha, Jamal Udin, K M Ishtiaque Sayeed, Denise O Garrett, Kashmira Date, Taiwo Abimbola

**Affiliations:** 1 Global Immunization Division, US Centers for Disease Control and Prevention (CDC), Atlanta, Georgia, USA; 2 Child Health Research Foundation, Dhaka, Bangladesh; 3 Applied Epidemiology, Sabin Vaccine Institute, Washington, DC, USA

**Keywords:** Typhoid, paratyphoid, enteric fever, cost of illness, Bangladesh

## Abstract

**Background:**

We conducted a cost of illness study to assess the economic burden of pediatric enteric fever (typhoid and paratyphoid) in Bangladesh. Results can inform public health policies to prevent enteric fever.

**Methods:**

The study was conducted at 2 pediatric health facilities in Dhaka. For the patient and caregiver’s perspective, we administered questionnaires on costs incurred from illness onset until the survey dates to caregivers of patients with blood culture positive cases at enrollment and 6 weeks later to estimate the direct medical, direct nonmedical, and indirect costs. From the perspective of the health care provider, we collected data on quantities and prices of resources used by the 2 hospitals to estimate the direct medical economic costs to treat a case of enteric fever. We collected costs in Bangladeshi takas and converted them into 2018 US dollars. We multiplied the unit cost per procedure by the frequency of procedures in the surveillance case cohort to calculate the average cost per case.

**Results:**

Among the 1772 patients from whom we collected information, the median cost of illness per case of enteric fever from the patient and caregiver perspective was US $64.03 (IQR: US $33.90 –$173.48). Median direct medical and nonmedical costs per case were 3% of annual labor income across the sample. From the perspective of the healthcare provider, the average direct medical cost per case was US $58.64 (range: US $37.25 at Hospital B, US $73.27 at Hospital A).

**Conclusions:**

Our results show substantial economic burden of enteric fever in Bangladesh, with higher costs for patients receiving inpatient care. As antimicrobial resistance increases globally, the cost of illness could increase, due to more expensive and potent drugs required for treatment.

In low- and middle-income tropical countries, enteric fever (typhoid and paratyphoid fevers) is a major public health problem due to low access to safe water, sanitation, and hygiene facilities. Globally, there are an estimated 10.9 million typhoid fever cases and 3.4 million paratyphoid fever cases per year [1], with a growing trend of antibiotic resistance in enteric fever pathogens in Asia and Africa [2]. The cost of illness (COI) due to enteric fever includes, as for any other disease, the direct costs to the health system and expenditures by patients and their families, as well as the indirect costs of missed work and school days, and in severe cases, death [3]. Estimating the economic burden of enteric fever is important to characterize the value of preventive interventions such as vaccination and improvements in water, sanitation, and hygiene. Such COI evidence is especially needed for decisions about introduction of new typhoid Vi-conjugate vaccines (TCVs), which the World Health Organization (WHO) recommended for routine use in endemic countries in 2017. The first of these vaccines (Typbar-TCV) was prequalified by WHO in early 2018 [2] and introduced for the first time to the routine immunization program of a country in Pakistan in 2019 [4].

Although previous literature has estimated the COI of enteric fever in other Asian countries, including India, Pakistan, Indonesia, Vietnam, China, and Nepal [5–8], no such study has been conducted in Bangladesh, the eighth most populous country in the world and a country in which enteric fever is highly endemic [9]. Moreover, except for a small-scale study in Nepal in 2015 and the other studies in Nepal and Pakistan in 2018 from the Surveillance for Enteric Fever in Asia Project (SEAP) II also included in this Supplement [10, 11], all prior studies were conducted over 15 years ago. Therefore, these estimates may no longer give an accurate picture of the economic burden, given rapid economic development and changes in health care landscape of countries in the region, including in Bangladesh. In addition, only 2 studies in the existent literature report results for pediatric populations, which represent more than half of the global cases (children ≤ 15 years were 56% of total cases in 2017 [12]). Previous literature has focused on patient costs and health facility costs for general outpatient and inpatient care, with limited detail on particular cost categories and specific procedures involved in enteric fever diagnosis and treatment. Past COI studies have also mainly focused on typhoid. With the increasing burden of paratyphoid and increasing antimicrobial resistance globally, it is important to understand the economic burden of paratyphoid and how treatment costs may be growing with the use of more potent and expensive drugs for both typhoid and paratyphoid.

A COI study of enteric fever with recent data from Bangladesh can fill this knowledge gap and be used by policy makers to estimate the cost-effectiveness and return on investment of preventing enteric fever, including consideration of different strategies to introduce TCV vaccines. Moreover, a comprehensive COI using the patient and caregiver and the health care provider perspectives is important to inform public policies targeting high risk groups in settings such as Bangladesh, where patient expenditures constitute an estimated 63% of total health expenditures, with greater relative economic burden on the poorest households [13], who may also be at higher risk of enteric fever due to environmental and socioeconomic factors [14, 15]. Accordingly, this study presents the first enteric fever COI estimates for pediatric populations in Bangladesh based on empirical data collection in a sample of sites from 2 perspectives: (i) patient and caregiver, and (ii) health care provider.

## METHODS

### Study Setting

This COI study was a prospective study coordinated with SEAP II, and conducted at 2 pediatric health facilities located in Dhaka (estimated population: 20 million [16]), the capital city of Bangladesh. Both are private not-for-profit hospitals with an annual patient volume of 270 792 for Hospital A and 295 753 for Hospital B, as of 2016. These health facilities were selected based on their laboratory capacity to perform blood culture testing for typhoid and paratyphoid, and previous involvement in enteric fever surveillance, and were not intended to be representative of Dhaka or Bangladesh.

### Cost of Illness from the Patient and Caregiver Perspective

#### Study Design

Patients eligible for enrollment in the health facility-based surveillance component were those ≤ 17 years with blood culture-confirmed cases of *Salmonella* (*S.*) Typhi or Paratyphi, or with a nontraumatic terminal ileal perforation, regardless of blood culture result. Participants in the COI component were patients enrolled through the 2 participating health facilities or through their laboratories.

This study included 3 types of costs incurred by the patient and their caregivers from illness onset until the survey dates: direct medical costs, direct nonmedical costs, and indirect costs. Direct medical costs included the monetary value of health facility registration fees, clinical examination, inpatient stay, laboratory tests, drugs and medications, and other diagnostic and treatment services (eg, X-ray, surgery). Direct nonmedical costs included transport, food, lodging, and care services for family members. Since the study sites were pediatric hospitals, patient indirect costs included only the days of school missed, which were not monetized. The indirect costs of caregivers included the value of work days missed and sick leave days taken to care for the patient, valued at the median of caregivers’ self-reported salary range (eg, if respondent indicated a salary in the range of 0–1600 Bangladeshi takas, the midpoint of this range—800 takas—was used to value their time). The value of the time spent by caregivers who did not routinely earn a wage (eg, unpaid household labor) only included the time spent at health facilities and was monetized exclusively in the sensitivity analysis.

Costs excluded were costs of nonenteric fever-related drugs and therapies such as antimalarial medications, costs of diagnosis and treatment of co-morbidities, and chronic conditions not directly related to enteric fever, intangible costs related to pain and suffering, and costs of services that the patient received at no charge at the health care facilities (eg, free inpatient care for the poor).

#### Data Collection

Cost questionnaires were developed in English and piloted in the sites, then translated into Bengali. Questionnaires were administered in Bengali by bilingual SEAP II interviewers, with data recorded electronically via tablet. Cost data were collected from September 2016 to December 2018 through telephone by the same SEAP II interviewers who administered the surveillance questionnaires. The pediatric patients’ caregivers responded to the cost questionnaires at 2 time points: (i) 2 to 3 days after laboratory testing or hospital discharge, and (ii) 6 weeks (approximately 42 days) after study enrollment. The first cost questionnaire collected information about the costs incurred from illness onset to the enrollment visit, while the second cost questionnaire collected costs incurred after the enrollment visit until the follow-up call.

### Cost of Illness Measures and Data Analysis

COI measures included median patient direct medical and nonmedical costs, median number of days of school lost by patients, median number of days of work lost and sick leave by caregivers, and the median productivity loss due to lost wages by the caregiver. COI from the patient and caregiver perspective for an episode of enteric fever was calculated as the sum of the direct medical costs, direct nonmedical costs, and indirect costs.

Costs were collected in local currency (Bangladeshi takas), adjusted to 2018 values based on inflation rates from the Central Bank of Bangladesh, and converted into 2018 US dollars using the annual average exchange rate for 2018 (83.47 takas per US dollar [17]). Missing wage information for caregivers reporting work days missed or sick leave days taken was imputed with the median wage of the sample.

Two sensitivity analyses were conducted: (i) without outliers from each category of patient and caregiver costs, and (ii) imputing the wage level with the median wage reported in the sample and with the minimum daily wage rate of 220.42 takas (or US $2.64) for the indirect costs of the caregiver who routinely does not earn an income [18]. Outliers were defined as the observations with values above or below 2.24 standard deviations from the mean [19]. COI results were also presented separated for patients with *S.* Typhi, and *S.* Paratyphi.

### Cost of Illness From the Health Care Provider Perspective

#### Study Design

COI from the health care provider perspective was estimated for the same SEAP II study pediatric hospitals and included the direct medical economic costs (ie, the value of all resources used, not only financial outlays) to the hospitals to treat a case of enteric fever regardless of the source of funding. Hospital-related data were collected for the 2015–2016 fiscal year.

The medical procedures normally used to diagnose and treat a case of enteric fever and its associated complications at these sites were classified into 2 types: (i) medical procedures for which resource use did not differ between patients with enteric fever and patients with other diseases (eg, outpatient visit), and (ii) medical procedures particular to enteric fever (eg, blood culture test for typhoid and paratyphoid). Top-down activity-based macro-costing was used to estimate the cost of procedures in the first category, namely: outpatient visits, inpatient bed days, emergency visits, neonatal intensive care unit bed days, outpatient surgery visits, and inpatient surgery bed days, as well as to allocate cross-cutting administrative services, utilities and communication, and clinical supportive services (eg, laundry and waste disposal). Bottom-up ingredients-based micro-costing was used to estimate the costs of procedures in the second category, such as blood draws, blood culture tests, Widal tests, complete blood counts tests, C-reactive protein tests, abdominal X-rays, abdominal ultrasounds, surgeries for intestinal perforation, and gallbladder surgeries; and to calculate the cost of the personnel in the procedures of the first category.

Cost categories included were personnel salaries, labor time, materials and supplies, equipment and instruments, contracted services, equivalent rental value of the building space, administrative services, and clinical support services (eg, laundry, cleaning, patient meals). Costs excluded were those associated with magnetic resonance imaging and computed tomography scans (as these were reportedly infrequently used for enteric fever diagnosis in the study sites); patient registration; medication costs paid for by patients (rather than by the hospital as part of procedure provision); costs of nonenteric fever-related drugs and therapies (eg, antimalarial treatment); costs of diagnosis and treatment of co-morbidities and chronic conditions not directly related to enteric fever; nonclinical costs (eg, teaching salaries and classroom space) for study sites that are also academic training centers; evaluation-specific costs; and value of study team staff time for project management, technical assistance, and evaluation.

#### Data Collection

The data collection tool was designed in Microsoft Excel and piloted in the sites. Data on prices and quantities of resources used, as well as service volumes, were collected in a paper-based version of the tool between March–August 2017 by 2 local SEAP research assistants, with technical assistance from CDC. Data sources were annual financial reports, administrative records from the accounting department and clinical wards, on-site observation, and interviews with administrative and medical staff. Data on the frequencies of the medical procedures conducted for the patients with blood culture-confirmed enteric fever or nontraumatic ileal perforation in these health facilities were collected in the surveillance component of the SEAP II study during the period September 2016 to December 2018. Missing prices of items in the various categories of supplies and materials and equipment and instruments were imputed with data from the UNICEF supply catalog [20], and, if unavailable from UNICEF, with price data from the other health facility. Supplies and materials whose prices were unavailable from other sources, but tended to be very low (eg, an envelope) remained as missing values.

### Cost of Illness Measures and Data Analysis

Data were analyzed in Microsoft Excel in 4 stages. First, for enteric fever-specific procedure costs estimated using ingredients-based micro-costing, the unit cost per clinical procedure was calculated based on the quantity of resources used in that procedure, multiplied by the monetary value of each resource:

$$=\underset{j=1}{\sum^{N}}(quantity\;of\;resource\;input\;used_{ij}\text{*}price\;of\;resource\;input_{j})$$

where ***i*** is the procedure, and ***j*** indexes each resource input used in the procedure up to ***N*** resources.

Second, for general procedure costs estimated using activity-based macro-costing, the monetary value of all resources (eg, equipment, supplies) except personnel used in that service ward over the fiscal year was divided by the service volume in that ward (eg, number of outpatient visits):

$$=\;\underset{j=1}{\sum^{N}}(quantity\;of\;resource\;input\;used_{wj}\text{*}price\;of\;resource\;input_{j})/{\left(service\;volume\right)}_{w}$$

where ***w*** is the ward and ***j*** indexes each resource input used in the ward up to ***N*** resources.

To complete the calculation of the unit cost per clinical procedure, a fraction of the hospital-level services costs had to be added to the micro-costing and macro-costing calculations. Thus, cross-cutting administrative and clinical supportive services were allocated evenly across all wards in the hospital, and then were divided by the service volume in each ward. The results were unit costs of administrative and clinical supportive services that were added to both general procedure cost and enteric fever-specific procedure cost.

In the final stage, the average direct medical cost per case of enteric fever was calculated by multiplying the unit cost per clinical procedure by the procedure’s frequency in the patient cohort of enteric fever cases identified through blood culture or nontraumatic ileal perforation from the surveillance study component, then summing these costs and dividing by the number of enteric fever cases from the surveillance component:

$$=\frac{\sum_{k=1}^{N}(health\;facility\;unit\;cost\;procedure_{i}\text{*}frequency\;of\;procedure_{ik})}{Total\;number\;of\;confirmed\;enteric\;fever\;cases\;(N)}$$

where ***i*** is the procedure, ***k*** indexes the confirmed enteric fever cases at the health facility, and ***K*** is the total number of confirmed enteric fever cases identified at the health facility during the surveillance study period.

Health facility costs were collected in local currency (Bangladeshi takas) and adjusted with the same inflation and exchange rates as the patient and caregiver COI costs.

#### Ethical Considerations

The study protocol was approved by the Bangladesh Institute of Child Health Ethical Review Committee. In accordance with the human subjects review procedures of the US Centers for Disease Control and Prevention (CDC), it was determined that the CDC was not formally engaged in human subjects research.

## RESULTS

### Cost of Illness From the Patient and Caregiver Perspective

#### Patient Characteristics

Of 2203 enrolled patients eligible for the COI interview, 1772 (80.4%) responded to the first cost questionnaire covering costs through the enrollment visit, and 1693 (76.8%) responded to the second cost questionnaire covering costs up to the six-week follow-up call (Table 1). Of the first questionnaire respondents, 13.1% were less than 2 years old, 38.2% were 2 to 4 years old, and 48.7% were 5 to 17 years old; 55% were male. There were 1530 blood culture-positive *S.* Typhi cases, 240 blood culture-positive *S.* Paratyphi cases, and 2 cases with blood culture-negative intestinal perforation (86.3%, 13.5%, and 0.1% respectively). Socioeconomically, most patients’ households had electricity (90.9%), cement roofs (82.3%), a household flush toilet (84.9%), a mobile phone (90.3%), and treated their drinking water at home (54.1% by boiling, 13.6% by other methods).

**Table 1.** Patient and Caregiver Cost of Illness Due to Enteric Fever, Sample Characteristics, Dhaka, Bangladesh, September 2016–December 2018

Characteristic	n	%
Respondents		
Patients enrolled in surveillance study	2203	100.0
Patients responding to enrollment cost questionnaire	1772	80.4
Patients responding to 6-week follow-up cost questionnaire	1693	76.8
Patients who died of enteric fever	0	0.0
Cost of illness patient sample: respondents to enrollment cost questionnaire only (n = 1772)		
Age (years)		
<2	232	13.1
2–4	677	38.2
5–17	863	48.7
Sex		
Male	975	55.0
Female	797	45.0
Blood culture result		
*Salmonella* Typhi positive	1530	86.3
*Salmonella* Paratyphi positive	240	13.5
Not positive for either *Salmonella* Typhi or Paratyphi (surgical cases)	2	0.1
Household with mobile phone		
Yes	1600	90.3
No	24	1.4
Did not respond	148	8.4
Households with electricity		
Yes	1611	90.9
No	13	0.7
Did not respond	148	8.4
Households with car/motorcycle		
Yes	87	4.9
No	1537	86.7
Did not respond	148	8.4
Household roof material		
Cement	1457	82.3
Metal sheets, mats, ceramic, shingles	313	17.6
Natural materials	2	0.1
Households with sanitation		
Household flush to sewer system, septic tank, somewhere else	1504	84.9
Household pit latrine, bucket or hanging toilet, communal toilet, other	120	6.8
Did not respond	148	8.4
Drinking water treated at home		
Boil	958	54.1
Chlorine liquid, powder, or tablets	100	5.6
Other	142	8.0
Do not treat water	149	8.4
Did not respond	423	23.9

#### Patient and Caregiver Direct Medical and Nonmedical Costs

**Figure 1.**
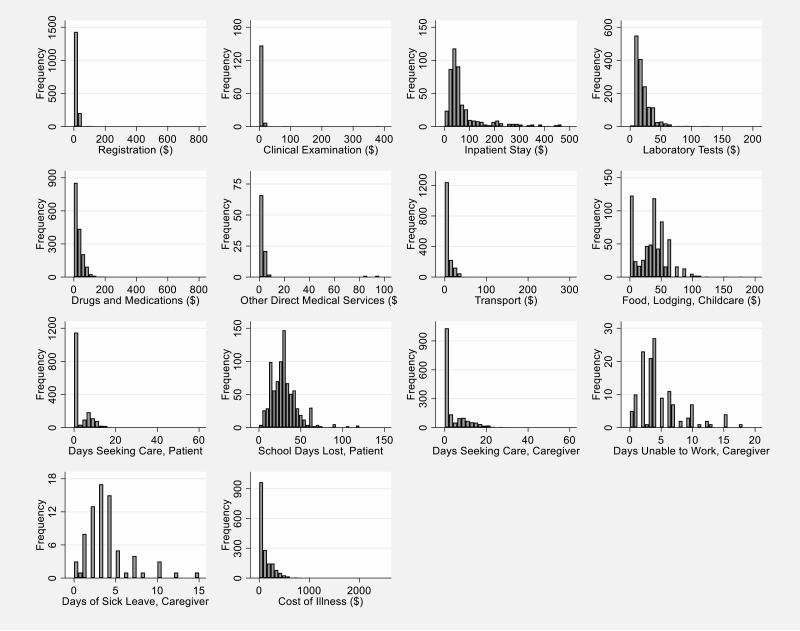
Distribution of cost of illness elements from the patient and caregiver perspective, Dhaka, Bangladesh, September 2016—December 2018 (N = 1772 patients, 2018 US $).

Of the 1772 patients, 1735 (97.9%) reported some direct medical costs (Table 2). Direct medical costs (median US $56.55, IQR: US $30.35–$139.08 for all patients) were the largest component of the COI for all patients and patient sub-samples (patients who reported inpatient care and patients who only reported outpatient care). Although not all respondents were able to recall the costs they had paid for specific procedures, the most frequently reported costs were registration (reported by 94.0%, median cost: US $1.44, IQR: US $0.72–$5.06), drugs and medications (reported by 93.2%, median cost: US $22.76; IQR: US $11.98–$43.46), and laboratory tests (reported by 88.2%, median cost: US $16.94; IQR: US $11.86-$25.29) (Figure 1). The largest proportion of the direct medical expenditure was for the treatment costs during inpatient care. For those patients reporting inpatient care costs, median costs were higher for each procedure type, reflecting a combination of more intensive and more expensive care.

**Table 2.** Patient and Caregiver Cost of Illness Due to Enteric Fever: Direct Medical, Direct Nonmedical, Indirect, and Total Costs, Dhaka, Bangladesh, September 2016—December 2018 (N = 1772)

	Patients who did not report any inpatient care expenses^a^				Patients who reported inpatient care expenses^a^				All patients			
Cost type	n	Median	25th Pctl	75th Pctl	n	Median	25th Pctl	75th Pctl	n	Median	25th Pctl	75th Pctl
Direct medical costs (2018 US $)												
Total direct medical costs	1267	40.46	25.29	65.89	470	195.28	143.76	284.49	1735	56.55	30.35	139.08
Registration	1220	1.20	0.72	2.88	446	10.18	3.59	29.59	1666	1.44	0.72	5.06
Clinical examination	94	5.06	3.79	7.19	62	6.32	5.06	8.39	156	5.99	4.79	8.39
Inpatient Stay	0	N/A	N/A	N/A	470	47.92	33.54	75.86	470	47.92	33.54	75.86
Laboratory tests	1157	14.98	10.78	20.37	406	29.95	19.77	37.93	1563	16.94	11.86	25.29
Drugs and medications	1208	16.44	10.12	29.95	444	51.68	36.67	72.07	1652	22.76	11.98	43.46
Other services^b^	67	2.52	2.52	3.59	24	2.52	1.26	2.53	91	2.52	1.68	3.59
Direct nonmedical costs (2018 US $)												
Total direct nonmedical costs	1213	3.35	2.28	6.32	465	53.91	38.58	70.44	1678	5.44	2.64	36.67
Transport	1207	3.16	2.16	5.69	444	13.35	7.59	23.96	1651	4.43	2.53	9.58
Food, lodging, childcare	207	5.06	2.53	29.95	442	37.93	29.95	50.58	649	35.94	15.17	48.05
Indirect costs—Patient												
Days spent seeking care	1236	0.21	0.17	0.38	470	7.00	6.00	9.00	1706	0.33	0.17	6.00
School days lost	611	27.00	17.00	34.00	207	33.00	23.00	43.00	818	29.00	18.00	36.00
Indirect costs—Caregiver												
Days spent accompanying the patient seeking care	1237	0.34	0.21	1.08	462	9.65	7.00	14.00	1699	1.00	0.25	7.00
Days unable to work	65	3.00	2.00	5.00	70	4.00	3.00	6.13	135	4.00	2.00	6.00
Days of sick leave	40	3.00	2.00	4.00	33	3.00	2.33	5.00	73	3.00	2.00	4.00
Total productivity loss (2018 US $)	104	19.57	12.58	26.55	98	25.16	17.60	39.83	202	19.92	13.28	33.19
Cost of illness (2018 US$)												
Total cost of illness per case	**1267**	**45.41**	**28.45**	**75.86**	**470**	**262.69**	**178.75**	**351.25**	**1737**	**64.03**	**33.90**	**173.48**
Total cost of illness per case without the value of sick leave	**1267**	**44.93**	**28.45**	**74.71**	**470**	**260.78**	**178.75**	**351.25**	**1737**	**63.26**	**33.89**	**172.59**

Abbreviations: N/A, not applicable because it excludes inpatient care; 25th Pctl, 25^th^ percentile; 75th Pctl, 75^th^ percentile.

^a^Inpatient care expenses: expenses on inpatient care regardless of patient recruitment location (outpatient care, inpatient care, hospital laboratory, surgery, laboratory network).

^b^Other services that patients did not report in the above categories (eg, medical materials or equipment for surgery).

Direct nonmedical costs while seeking and receiving care, such as transport, food, lodging, and child care, were reported by 1678 patients (94.7% of total sample), with a median cost of US $5.44 (IQR: US $2.64-$36.67) (Table 2). Of those reporting any nonmedical direct costs, the most frequently reported was for transport (reported by 98.4%, median cost: US $4.43; IQR: US $2.53–9.58) (Figure 1). Only 649 patients (36.7%) reported other nonmedical costs for food, lodging, child care, or other expenses, though at a higher median cost of US $35.94 (IQR: US $15.17–$48.05). Direct nonmedical costs of patients reporting inpatient care were higher in all cases.

#### Patient and Caregiver Indirect Costs

In this pediatric patient sample, 818 patients reported a median of 29.00 school days lost (IQR: 18.00–36.00 days) (Table 2 and Figure 1). There were 135 patients whose caregivers were unable to work for a median of 4.00 days (IQR: 2.00–6.00 days) and 73 patients whose caregivers used a median of 3.00 days of sick leave (IQR: 2.00–4.00 days). When valued at the median of caregivers’ reported wage rate ranges, these days of productive work lost, plus paid sick leave used, translated into a productivity loss of US $19.92 (IQR: US $13.28–$33.19) per case. On average, each patient received the care of 1.9 caregivers. In all sub-categories, productivity loss was higher for patients reporting inpatient care.

#### Median Cost Per Case of Enteric Fever

After adding direct medical costs, direct nonmedical costs, and indirect costs of caregiver productivity losses, the median cost of illness per case of enteric fever from the patient and caregiver perspective was US $64.03 (IQR: US $33.90–$173.48) for all patients (Table 2 and Figure 1). Median COI for patients receiving inpatient care was almost 6 times higher than the COI of patients only reporting outpatient care. Furthermore, when removing the value of the sick leave (potentially borne by the employers and not by the patient and caregivers), the median COI decreased marginally to US $63.26 (IQR: US $33.89–$172.59).

#### Sensitivity Analysis

Results changed minimally as expected when removing outlier values (median COI slightly decreased from US $64.03 to $61.32), imputing wages for unpaid labor (higher median COI of US $74.16 for all patients), and using the national minimum wage rates for caregivers (lower median COI of US $70.22 for all patients) (Tables 3 and 4). Results were similar for patients with typhoid or paratyphoid fever, with higher median costs for paratyphoid patients in some sub-categories but lower median COI overall (US $59.43) compared with typhoid patients (US $64.69) (Tables 5 and 6). In addition, our sample contained 2 cases of patients with nontraumatic terminal ileal gastrointestinal perforations which were not lab-confirmed for enteric fever (potentially unrelated to enteric fever). After removing the 2 cases of patients with nontraumatic terminal ileal gastrointestinal perforations, the median COI for all patients decreased by cents to US $63.97 (IQR: US $33.90 –$172.59, data not shown).

**Table 3.** Sensitivity Analysis of Patient and Caregiver Cost of Illness Due to Enteric Fever: Direct Medical, Direct Nonmedical, Indirect, and Total Costs When Excluding Outliers, Dhaka, Bangladesh, September 2016—December 2018

	Patients who did not report any inpatient care expenses^a^				Patients who reported inpatient care expenses^a^				All patients			
Cost type	n	Median	25th Pctl	75th Pctl	n	Median	25th Pctl	75th Pctl	n	Median	25th Pctl	75th Pctl
Direct medical costs (2018 US $)												
Total direct medical costs	1263	39.77	25.29	65.53	424	189.66	128.19	244.56	1687	53.91	29.97	126.44
Registration	1215	1.20	0.72	2.88	423	8.39	3.59	29.59	1638	1.44	0.72	4.79
Clinical examination	92	5.06	3.79	7.19	62	6.32	5.06	8.39	154	5.99	4.79	8.39
Inpatient Stay	0	N/A	N/A	N/A	444	45.52	31.15	70.81	444	45.52	30.75	70.81
Laboratory tests	1149	14.98	10.78	20.25	362	26.57	18.97	35.94	1511	16.77	11.86	24.02
Drugs and medications	1203	16.44	10.12	29.95	414	49.81	35.94	67.09	1617	21.56	11.98	41.93
Other services^b^	67	2.52	2.52	3.59	22	1.89	1.26	2.53	89	2.52	1.68	3.35
Direct nonmedical costs (2018 US $)												
Total direct nonmedical costs	1207	3.35	2.16	6.32	414	50.75	36.20	65.29	1621	5.06	2.64	29.95
Transport	1201	3.16	2.16	5.69	410	12.14	7.08	20.61	1611	4.31	2.40	8.85
Food, lodging, childcare	205	5.06	2.53	29.35	431	37.93	29.95	50.58	636	35.94	14.38	47.92
Indirect costs—Patient												
Days spent seeking care	1225	0.21	0.17	0.38	437	7.00	5.13	8.13	1662	0.29	0.17	5.00
School days lost	605	27.00	17.00	33.00	194	30.00	22.00	40.00	799	28.00	18.00	36.00
Indirect costs—Caregiver												
Days spent accompanying the patient seeking care	1223	0.34	0.21	1.08	407	9.00	6.38	12.08	1630	0.58	0.23	6.00
Days unable to work	62	3.00	2.00	5.00	67	4.00	3.00	6.00	129	4.00	2.00	6.00
Days of sick leave	39	3.00	2.00	4.00	29	3.00	2.00	4.00	68	3.00	2.00	4.00
Total productivity loss (2018 US $)	102	19.04	12.58	26.55	92	25.16	16.51	37.74	194	19.92	13.28	31.45
Cost of illness (2018 US $)												
Total cost of illness per case	1263	45.17	28.45	75.36	418	246.56	170.60	317.48	1681	61.32	33.31	157.90

Abbreviations: N/A, not applicable because it excludes inpatient care; 25th Pctl, 25^th^ percentile; 75th Pctl, 75^th^ percentile.

^**a**^Inpatient care expenses: expenses on inpatient care regardless of patient recruitment location (outpatient care, inpatient care, hospital laboratory, surgery, laboratory network).

^b^Other services that patients did not report in the above categories (eg, medical materials or equipment for surgery).

**Table 4.** Sensitivity Analysis of Patient and Caregiver Cost of Illness Due to Enteric Fever: Indirect and Total Costs When Using Alternative Wage Rates and Including Unpaid Labor, Dhaka, Bangladesh, September 2016—December 2018 (N = 1772, 2018 US $)

	Patients who did not report any inpatient care expenses^a^				Patients who reported inpatient care expenses^a^				All patients			
Cost type	n	Median	25th Pctl	75th Pctl	n	Median	25th Pctl	75th Pctl	n	Median	25th Pctl	75th Pctl
Indirect costs—Caregiver—using median wage in the sample for unpaid caregivers’ time												
Total productivity loss	1237	2.18	1.31	7.86	462	63.75	44.16	94.35	1699	6.29	1.57	44.56
Cost of illness - using median wage in the sample for unpaid caregivers’ time												
Total cost of illness per case	**1269**	**49.63**	**31.67**	**85.61**	**470**	**357.55**	**268.27**	**475.92**	**1739**	**74.16**	**37.14**	**251.72**
Indirect costs—Caregiver—using country minimum wage for unpaid caregivers’ time												
Total productivity loss	1237	0.92	0.55	3.30	462	29.05	18.71	43.39	1699	2.64	0.66	21.16
Cost of illness—using country minimum wage for unpaid caregivers’ time												
Total cost of illness per case	**1269**	**45.31**	**30.54**	**81.83**	**470**	**321.96**	**234.49**	**426.84**	**1739**	**70.22**	**35.80**	**221.45**

Abbreviations: 25th Pctl, 25^th^ percentile; 75th Pctl, 75^th^ percentile.

^a^Inpatient care expenses: expenses on inpatient care regardless of patient recruitment location (outpatient care, inpatient care, hospital laboratory, surgery, laboratory network).

**Table 5.** Patient and Caregiver Cost of Illness Due to Blood Culture-confirmed Typhoid Fever: Direct Medical, Direct Nonmedical, Indirect, and Total Costs, Dhaka, Bangladesh, September 2016—December 2018 (N = 1530)

	Patients who did not report any inpatient care expenses ^a^				Patients who reported inpatient care expenses ^a^				All patients			
Cost type	n	Median	25th Pctl	75th Pctl	n	Median	25th Pctl	75th Pctl	n	Median	25th Pctl	75th Pctl
Direct medical costs (2018 US $)												
Total direct medical costs	1084	39.60	25.29	66.08	416	194.68	143.76	281.07	1500	56.90	30.35	142.39
Registration	1041	1.20	0.72	2.53	394	11.02	3.71	29.59	1435	1.44	0.72	5.06
Clinical examination	83	5.06	3.79	7.59	50	6.32	5.06	8.39	133	5.99	4.79	8.39
Inpatient stay	0	N/A	N/A	N/A	416	47.92	33.54	75.86	416	47.92	33.54	75.86
Laboratory tests	988	14.67	10.78	20.37	358	27.82	18.97	37.93	1346	16.77	11.86	25.29
Drugs and medications	1032	16.29	10.12	29.95	394	51.13	36.67	72.07	1426	22.76	11.98	43.55
Other services^b^	59	2.52	2.52	3.59	19	1.26	1.26	2.53	78	2.52	1.68	3.59
Direct nonmedical costs (2018 US $)												
Total direct nonmedical costs	1035	3.23	2.16	5.99	411	53.91	39.20	70.44	1446	5.39	2.64	38.34
Transport	1029	3.16	2.16	5.69	392	13.23	7.19	22.76	1421	4.43	2.40	9.58
Food, lodging, childcare	169	5.06	2.53	29.95	392	38.43	29.95	50.58	561	35.94	17.97	48.05
Indirect costs—Patient												
Days spent seeking care	1055	0.21	0.17	0.38	416	7.00	6.00	9.00	1471	0.33	0.17	6.00
School days lost	514	27.00	17.00	34.00	182	34.00	24.00	44.00	696	29.00	18.00	36.00
Indirect costs—Caregiver												
Days spent accompanying the patient seeking care	1058	0.34	0.21	1.08	409	10.00	7.03	14.01	1467	1.00	0.25	7.04
Days unable to work	50	3.00	2.00	5.00	62	4.00	3.00	7.00	112	4.00	2.00	6.00
Days of sick leave	33	3.00	1.00	4.00	27	3.00	2.33	5.00	60	3.00	2.00	4.00
Total productivity loss (2018 US $)	83	19.22	12.58	26.55	85	25.16	17.60	39.83	168	21.96	13.28	37.74
Cost of illness (2018 US $)												
Total cost of illness per case	1084	44.69	27.82	75.17	416	260.90	183.14	351.12	1500	64.69	33.66	177.02

Abbreviations: N/A, not applicable because it excludes inpatient care; 25th Pctl, 25^th^ percentile; 75th Pctl, 75^th^ percentile.

^a^Inpatient care expenses: expenses on inpatient care regardless of patient recruitment location (outpatient care, inpatient care, hospital laboratory, surgery, laboratory network).

^b^Other services that patients did not report in the above categories (eg, medical materials or equipment for surgery).

**Table 6.** Patient and Caregiver Cost of Illness Due to Blood Culture-confirmed Paratyphoid Fever: Direct Medical, Direct Nonmedical, Indirect, and Total Costs, Dhaka, Bangladesh, September 2016—December 2018 (N = 240)

	Patients who did not report any inpatient care expenses^a^				Patients who reported inpatient care expenses^a^				All patients			
Cost type	n	Median	25th Pctl	75th Pctl	n	Median	25th Pctl	75th Pctl	n	Median	25th Pctl	75th Pctl
Direct medical costs (2018 US $)												
Total direct medical costs	183	42.99	27.14	63.22	52	196.30	128.97	300.30	235	50.58	30.98	107.47
Registration	179	1.26	0.72	3.79	50	5.06	2.02	28.87	229	1.52	0.76	5.06
Clinical examination	11	5.99	5.06	6.32	12	8.39	5.39	12.31	23	6.32	5.06	8.39
Inpatient Stay	0	N/A	N/A	N/A	52	50.58	31.95	79.40	52	50.58	31.95	79.40
Laboratory tests	169	15.17	10.75	21.49	47	35.94	26.55	44.25	216	17.70	11.86	27.26
Drugs and medications	176	16.77	10.53	31.61	48	51.84	35.40	66.42	224	21.49	11.98	41.51
Other services^b^	8	2.52	1.98	3.15	3	2.53	1.26	2.53	11	2.52	1.68	2.53
Direct nonmedical costs (2018 US $)												
Total direct nonmedical costs	178	3.79	2.53	7.59	52	56.27	34.14	69.51	230	5.99	3.00	24.78
Transport	178	3.57	2.53	6.32	50	13.51	8.85	25.29	228	4.55	2.78	9.47
Food, lodging, childcare	38	3.79	2.53	25.29	49	37.93	29.95	53.11	87	30.35	4.79	47.92
Indirect costs—Patient												
Days spent seeking care	181	0.25	0.17	0.46	52	7.00	4.65	8.00	233	0.33	0.17	4.00
School days lost	97	27.00	16.00	32.00	25	30.00	22.00	35.00	122	27.00	17.00	33.00
Indirect costs—Caregiver												
Days spent accompanying the patient seeking care	180	0.42	0.22	1.17	52	9.04	6.00	13.52	232	1.00	0.26	4.71
Days unable to work	16	3.00	2.00	5.00	10	3.50	3.00	4.13	26	3.00	2.00	5.00
Days of sick leave	8	3.00	2.00	3.50	6	4.00	2.00	5.00	14	3.00	2.00	4.00
Total productivity loss (2018 US $)	23	18.87	12.58	28.98	15	19.92	18.87	33.19	38	19.92	13.28	28.98
Cost of illness (2018 US $)												
Total cost of illness per case	183	48.52	31.87	77.87	52	277.17	171.33	350.49	235	59.43	36.90	154.26

Abbreviations: N/A, not applicable because it excludes inpatient care; 25th Pctl, 25^th^ percentile; 75th Pctl, 75^th^ percentile.

^a^Inpatient care expenses: expenses on inpatient care regardless of patient recruitment location (outpatient care, inpatient care, hospital laboratory, surgery, laboratory network).

^b^Other services that patients did not report in the above categories (eg, medical materials or equipment for surgery).

### Cost of Illness from the Health Provider Perspective

Procedure costs varied across the 2 hospital sites because of differences in resource quantities and prices, organizational structure, and service volumes. The costliest procedures at both hospitals were gallbladder surgery (US $104.56 and $131.56; at Hospital A and Hospital B, respectively) and intestinal perforation surgery (US $94.99 and $119.85), while the least costly were blood draw (US $1.11 and $0.81) and selected laboratory tests (C-reactive protein at Hospital A: US $2.01; Widal test at Hospital B: US $1.50) (Table 7). Data on the frequencies of some specific procedures were not available from the surveillance component. The 1640 patients included in the frequency calculations were those < 18 years of age enrolled at the 2 hospital sites and laboratories, whether or not they consented to participate in the patient COI component. The frequency-weighted average direct medical cost per case of enteric fever was US $58.64 (range: US $37.25 at Hospital B, US $73.27 at Hospital A).

**Table 7.** Health Care Provider Cost of Illness Due to Enteric Fever: Procedure Unit Costs and Frequencies, and Average Cost Per Case of Enteric Fever, Dhaka, Bangladesh, July 2015—June 2016 (n = 1640)

	Unit cost in 2018 US$		Frequency	
Procedure	Hospital A	Hospital B	Hospital A	Hospital B
General services not specific to enteric fever				
Outpatient routine service cost (per patient, per visit)	$12.88	$1.30	535	391
Inpatient hospital cost (per patient, per day)	$13.32	$7.93	3266	1732
Surgical outpatient visit (per patient, per visit)	$10.75	N/A	2	N/A
Surgical inpatient (per patient, per day)	$14.15	$1.14	0	0
Neonatal ICU (per patient, per day)	$37.84	$8.48	...a	...a
Emergency routine service visit (per patient, per visit)	$14.32	$2.25	...a	...a
Services specific to enteric fever				
Gallbladder surgery	$104.56	$131.56	...a	...a
Surgery for intestinal perforation	$94.99	$119.85	2	0
Blood culture	$16.36	$11.93	974	666
Abdominal ultrasound^b^	$6.03	$10.51	84	7
Widal test	$5.10	$1.50	...a	...a
Complete blood count	$4.57	$4.62	644	383
Abdominal X-ray	$3.49	$4.91	102	49
C-reactive protein	$2.01	$12.40	...a	...a
Blood draw	$1.11	$0.81	917	660
Total blood culture-confirmed enteric fever or nontraumatic ileal perforation cases			974	666
Weighted average cost per case by hospital	$73.27	$37.25		
Weighted average cost per case (both hospitals)	$58.64			

Abbreviations: ICU, intensive care unit; N/A, not applicable, the hospital does not offer that service.

^a^Missing information.

^b^Based on number of chest x-rays as a proxy, as number of abdominal x-rays was not collected in the SEAP II clinical surveillance component.

## Discussion

Compared to previously published COI studies of typhoid and/or enteric fever in children in all countries, our results from Bangladesh show higher direct medical and nonmedical COI to patients and their families (Table 8) [5, 7]. However, within our SEAP country studies, which captured costs over a shorter follow-up period, the direct medical and nonmedical COI for Bangladesh was higher when compared with Nepal and lower compared with Pakistan [10, 11]. The lower indirect costs in our study reflect the monetization only of the productivity losses of caregivers, not the pediatric patients themselves. All costs and methods are otherwise similar, although the types of health facilities and organization of health care differ across countries, illustrating the importance of context-specific COI estimates. The higher magnitude of patient costs for our study in Bangladesh, compared to past studies for which data were collected in 1995–2003, may also reflect changes in the economy and higher health care prices today, indicating the relevance of updated COI estimates rather than merely adjusting past estimates for inflation for cost-effectiveness analyses or other modeling.

**Table 8.** Average Costs for a Case of Enteric Fever for Children and Adults in Bangladesh and Other Countries (2018 US $)

	SEAP			Other Studies					
	Bangladesh	Nepal [10]	Pakistan [11]	Tanzania [6]^a^	Vietnam [7]^b^	China [7]^b^	Indonesia [7]	Pakistan [7]^c^	India [7]
**Children (<18 years)**									
Direct (medical and non-medical) ($)	$123.47	$119.11	$362.95		$59.34	$99.20	$43.69	$64.98	$8.17
Indirect (days of work/sick leave/school lost)	13.87	20.14	22.17						
Indirect cost of caregivers ($)	$3.14	$11.53	$24.84	$172.65	$8.99	$17.40	$33.49	$13.00	$4.90
**Characteristics of studies**									
Year of data collection	2016–2018			2010	2002	2001	2002	2001	2003
Perspective	Patient and caregiver			Societal	Patient and provider				
Type of enteric fever	Typhoid and paratyphoid			Typhoid	Typhoid				
Period of costs included	Six weeks after enrollment in study			90 days after enrollment in study	90 days after onset of illness				
Costs included	Cost incurred by patient and caregiver			Cost incurred by patient and caregiver	Private cost incurred by patient and caregiver and public cost incurred by public hospitals free of charge for the patient				

Results from other countries were adjusted by inflating local currencies using local inflation rates and then exchanging to US dollars. The studies in this table have methodological differences that prevent them to be directly comparable. Source: references [6, 7, 10, 11].

^a^In this study children are ≤15 years.

^b^In this study children are 5–17 years.

^c^In this study children are 2–15 years.

Our results show that the potential economic burden of enteric fever to patients and their caregivers, as well as to health care providers, can be substantial in Bangladesh. Median direct medical costs to patients and caregivers due to enteric fever represented 152% of Bangladesh’s health expenditure per capita of US $37.10 (2016 value in 2018 US dollars [16, 21]), and 526% for patients reporting inpatient care expenses. From the provider perspective, the average cost per case of enteric fever represented 164% of health expenditure per capita. When compared to median wage rates reported in the caregiver sample extrapolated annually, median direct medical and nonmedical costs per case are 3% of annual individual income across the sample and 14% of annual individual income for patients reporting inpatient care expenses. Previous literature defined catastrophic health expenditures as COI that exceeded 10% of annual household income [22–24]. Sensitivity analyses were conducted to estimate productivity losses under different wage assumptions to address missing wage data and possible reporting bias around wages. Furthermore, increasing antimicrobial resistance may increase the economic burden of enteric fever by requiring more expensive drugs for treatment. For example, during the outbreak of extensively drug-resistant (XDR) typhoid fever in Pakistan between 2016 and 2018, the patient direct medical costs of XDR patients were at least twice as high than for non-XDR patients [25].

### Limitations

Our results are subject to several limitations. The patient and site samples are not representative of Bangladesh, nor of Dhaka. Although the number of patients is much larger than in previous studies, all cases are pediatric patients that sought care in urban private hospitals. Thus, care should be taken when using these results to estimate economic burden for different populations. Interviews were conducted by phone instead of in person, which could have affected the response rate (80.4% of eligible patients and 76.9% for the 6-week follow-up questionnaire). Patient and caretaker costs may also be subject to recall or reporting biases for direct expenses and time spent seeking and receiving care. A control group to account for potential background patient morbidity and health care costs was not included. The risks of increasing antimicrobial-resistant enteric fever and its associated costs were not modeled. For health care provider costs, some missing data (mainly medical supply prices) had to be imputed based on third party sources (eg, UNICEF), which may have resulted in higher or lower estimated costs. In addition, the costs of personnel time per procedure may be subject to recall or reporting biases.

Costs were not combined across perspectives due to the limited health facility sample that did not represent all health facilities visited by patients at which patient and caregiver costs were incurred. Also, there was limited ability of questionnaire respondents to recall and report expenses for specific clinical procedures (eg, by specific type of lab test) to match these with health facility costs. Thus, although related, the COI from the patient and caregiver perspective includes at least one health facility per patient and is larger than the COI from the provider perspective, which only includes the average cost of treating a patient in one health facility.

## Conclusions

Quantifying the economic burden of enteric fever to patients, caregivers, and health care providers can provide evidence of the economic value of preventive interventions, such as typhoid vaccines and improvements to water and sanitation. While recognizing the challenge of limited laboratory facilities for blood culture confirmation, future research on the economic burden of enteric fever would benefit from expanding the types and locations of health facilities included to obtain more representative costs at different levels of the health system, and from a diversity of geographic areas and patient populations in which epidemiology and care seeking patterns may vary. Further methodological efforts to integrate costs paid by patients and caregivers with those incurred by health care providers and other payers (eg, government) would also contribute to a fuller societal perspective estimate of the economic burden of enteric fever.

## Notes

***Acknowledgments.*** The authors acknowledge the support provided by the SEAP Bangladesh team, especially Maksuda Islam, Shampa Saha, Raktim Das, Sabbir Ahmed, and Abrar Ahmed; personnel in the SEAP Bangladesh study sites; other members of the SEAP project team who supported data collection and management, especially Ashley Tate Longley and Caitlin Barkume; and personnel at the International Centre for Diarrhoeal Disease Research, Bangladesh.

***Disclaimer.*** The results and conclusions in this article are those of the authors and do not necessarily represent the official position or policies of the US Centers for Disease Control and Prevention or other partners.

***Financial support.*** This work was supported by funding from the Bill & Melinda Gates Foundation (grant number INV-008335) and the United States Centers for Disease Control and Prevention.

***Supplement sponsorship.*** This supplement is sponsored by the Sabin Vaccine Institute and made possible by a grant from the Bill & Melinda Gates Foundation.

***Potential conflicts of interest.*** Dr Saha reports grants from the World Health Organization and the Bill & Melinda Gates Foundation during the conduct of the study, as well as grants from the World Health Organization, Bill & Melinda Gates Foundation, GlaxoSmithKline, Pfizer, Sanofi Pasteur, and Edinburgh University outside of the submitted work. K. D., T. A., S. P., and N. M. report a conditional gift agreement between the Sabin Vaccine Institute (Primary grantee for the Surveillance for Enteric Fever in Asia Project grant from the Bill and Melinda Gates Foundation) and the CDC Foundation. All other authors have no potential conflicts. All authors have submitted the ICMJE Form for Disclosure of Potential Conflicts of Interest. Conflicts that the editors consider relevant to the content of the manuscript have been disclosed.
